# A Collaborative Epidemiological Investigation into the Criminal Fake Artesunate Trade in South East Asia 

**DOI:** 10.1371/journal.pmed.0050032

**Published:** 2008-02-12

**Authors:** Paul N Newton, Facundo M Fernández, Aline Plançon, Dallas C Mildenhall, Michael D Green, Li Ziyong, Eva Maria Christophel, Souly Phanouvong, Stephen Howells, Eric McIntosh, Paul Laurin, Nancy Blum, Christina Y Hampton, Kevin Faure, Leonard Nyadong, C. W. Ray Soong, Budiono Santoso, Wang Zhiguang, John Newton, Kevin Palmer

**Affiliations:** 1 Centre for Tropical Medicine, Nuffield Department of Clinical Medicine, University of Oxford, Churchill Hospital, Oxford, United Kingdom; 2 Wellcome Trust—Mahosot Hospital-Oxford Tropical Medicine Research Collaboration, Mahosot Hospital, Vientiane, Lao People's Democratic Republic; 3 School of Chemistry and Biochemistry, Georgia Institute of Technology, Atlanta, Georgia, United States of America; 4 Intellectual Property Crime Unit, International Criminal Police Organization (INTERPOL), Lyon, France; 5 GNS Science, Lower Hutt, New Zealand; 6 Division of Parasitic Diseases, National Center for Infectious Diseases, Centers for Disease Control and Prevention, Atlanta, Georgia, United States of America; 7 Intellectual Property Division, Economic Crime Investigation Department, Ministry of Public Security, Beijing, People's Republic of China; 8 Western Pacific Regional Office of the World Health Organization, Manila, The Philippines; 9 United States Pharmacopeia, Rockville, Maryland, United States of America; 10 Therapeutic Goods Administration, Government of Australia, Symonston, Canberra, Australia; 11 Royal Canadian Mounted Police Forensic Laboratory Services, National Anti- Counterfeiting Bureau, Ottawa, Ontario, Canada; Cornell University, United States of America

## Abstract

**Background:**

Since 1998 the serious public health problem in South East Asia of counterfeit artesunate, containing no or subtherapeutic amounts of the active antimalarial ingredient, has led to deaths from untreated malaria, reduced confidence in this vital drug, large economic losses for the legitimate manufacturers, and concerns that artemisinin resistance might be engendered.

**Methods and Findings:**

With evidence of a deteriorating situation, a group of police, criminal analysts, chemists, palynologists, and health workers collaborated to determine the source of these counterfeits under the auspices of the International Criminal Police Organization (INTERPOL) and the Western Pacific World Health Organization Regional Office. A total of 391 samples of genuine and counterfeit artesunate collected in Vietnam (75), Cambodia (48), Lao PDR (115), Myanmar (Burma) (137) and the Thai/Myanmar border (16), were available for analysis. Sixteen different fake hologram types were identified. High-performance liquid chromatography and/or mass spectrometry confirmed that all specimens thought to be counterfeit (195/391, 49.9%) on the basis of packaging contained no or small quantities of artesunate (up to 12 mg per tablet as opposed to ∼ 50 mg per genuine tablet). Chemical analysis demonstrated a wide diversity of wrong active ingredients, including banned pharmaceuticals, such as metamizole, and safrole, a carcinogen, and raw material for manufacture of methylenedioxymethamphetamine (‘ecstasy'). Evidence from chemical, mineralogical, biological, and packaging analysis suggested that at least some of the counterfeits were manufactured in southeast People's Republic of China. This evidence prompted the Chinese Government to act quickly against the criminal traders with arrests and seizures.

**Conclusions:**

An international multi-disciplinary group obtained evidence that some of the counterfeit artesunate was manufactured in China, and this prompted a criminal investigation. International cross-disciplinary collaborations may be appropriate in the investigation of other serious counterfeit medicine public health problems elsewhere, but strengthening of international collaborations and forensic and drug regulatory authority capacity will be required.

## Abstract 2

Paul Newton and colleagues' international, collaborative study found evidence that counterfeit artesunate was being manufactured in China, which prompted a criminal investigation.

## Editors' Summary

**Background:**

Malaria is one of the world's largest public health problems, causing around 500 million cases of illness and at least one million deaths per year (the estimates vary widely). The most serious form of malaria is caused by the parasite Plasmodium falciparum, which has become resistant to multiple drugs that had previously been the cornerstones of antimalarial regimens. One group of drugs for treating malaria, the artemisinin therapies including artesunate, are based upon a Chinese herb called qinghaosu; these have now become vital to the treatment of P. falciparum malaria. But counterfeit artesunate, containing none or too little (“subtherapeutic levels”) of the active ingredient, is a growing problem especially in South and East Asia. Fake artesunate is devastating for malaria control: it causes avoidable death, reduces confidence in the drug, and takes away profit from legitimate manufacturers. Of major concern also is the potential for subtherapeutic counterfeit artesunate to fuel the parasite's resistance to the artemisinin group of drugs.

Previous estimates have suggested that between 33% and 53% of artesunate tablets in mainland South East Asia are counterfeit. In this paper the authors report on an unprecedented international collaboration and criminal investigation that attempted to quantify and source counterfeit artesunate among some of the most malarious countries in Asia.

**Why Was This Study Done?:**

Previous reports have identified the problem of fake artesunate, but as of yet there have been few reports on the potential solutions. Concerned health workers and scientists, the regional World Health Organization (WHO) office and the International Criminal Police Organization (INTERPOL) got together to discuss what could be done in May 2005 when it became clear the counterfeit artesunate situation was worsening in the Greater Mekong Sub-Region of South East Asia (comprising Cambodia, Lao People's Democratic Republic, Myanmar, Thailand, Vietnam, and Yunnan Province in the People's Republic of China). Their subsequent investigation combined the goals and methods of a range of concerned parties—police, scientists, and health workers—to identify the source of counterfeit artesunate in South East Asia and to supply the evidence to help arrest and prosecute the perpetrators.

**What Did the Researchers Do and Find?:**

The researchers conducted forensic analyses of samples of genuine and counterfeit artesunate. They selected these samples from larger surveys and investigations that had been conducted in the region beginning in the year 2000. Genuine samples were supplied by a manufacturer to provide a comparator. The authors examined the physical appearance of the packages and subjected the tablets to a wide range of chemical and biological tests that allowed an analysis of the components contained in the tablets.

When comparing the collected packages and tablets against the genuine samples, the researchers found considerable diversity of fake artesunate in SE Asia. Sixteen different fake hologram types (the stickers contained on packages meant to identify them as genuine) were found. Chemical analysis revealed that all tablets thought to be fake contained no or very small quantities of artesunate. Other ingredients found in the artesunate counterfeit tablets included paracetamol, antibiotics, older antimalarial drugs, and a range of minerals, and there were a variety of gases surrounding the tablets inside the packaging. Biological analyses of pollen grains inside the packaging suggested that the packages originated in the parts of South East Asia along the Chinese border.

**What Do these Findings Mean?:**

The results were crucial in helping the authorities establish the origin of the fake artesunate. For example, the authors identified two regional clusters where the counterfeit tablets appeared to be coming from, thus flagging a potential manufacturing site or distribution network. The presence of wrong active pharmaceutical ingredients (such as the older antimalarial drugs) suggested the counterfeiters had access to a variety of active pharmaceutical ingredients. The presence of safrole, a precursor to the illicit drug ecstasy, suggested the counterfeits may be coming from factories that manufacture ecstasy. And the identification of minerals indigenous to certain regions also helped identify the counterfeits' origin. The researchers concluded that at least some of the counterfeit artesunate was coming from southern China. The Secretary General of INTERPOL presented the findings to the Chinese government, which then carried out a criminal investigation and arrested individuals alleged to have produced and distributed the counterfeit artesunate.

The collaboration between police, public health workers and scientists on combating fake artesunate is unique, and provides a model for others to follow. However, the authors note that substantial capacity in forensic analysis and the infrastructure to support collaborations between these different disciplines are needed.

**Additional Information.:**

Please access these Web sites via the online version of this summary at http://dx.doi.org/10.1371/journal.pmed.0050032.

- The World Health Organization in 2006 created IMPACT—International Medical Products Anti-Counterfeiting Taskforce—with the aim of forging international collaboration to seek global solutions to this global challenge and in raising awareness of the dangers of counterfeit medical products. The task force membership includes international organizations, nongovernmental organizations, enforcement agencies, pharmaceutical manufacturers' associations, and drug and regulatory authorities. IMPACT's Web site notes that trade in counterfeit medicines is widespread and affects both developed and developing countries but is more prevalent in countries that have weak drug regulatory systems, poor supply of basic medicines, unregulated markets, high drug prices and/or significant price differentials. IMPACT holds international conferences and maintains a rapid alert system for counterfeit drugs.
- The drug industry's anticounterfeit organization, Pharmaceutical Security Institute, works to develop improved systems to identify the extent of the counterfeiting problem and to assist in coordinating international inquiries. Its membership includes 21 large pharmaceutical companies.
- The Web site of David Pizzanelli, a world expert on security holography, contains a PowerPoint presentation co-authored by Paul Newton that illustrates the different types of fake holograms found on fake artesunate packages, and their implications for artemisinin resistance (http://www.pizzanelli.co.uk/content/artesunate.html).

## Introduction

Malaria still claims over one million lives each year and is a risk for some 40% of the world's population [1,2]. Most patients with malaria would survive if they had timely access to efficacious medicines that they could afford. With the global spread of drug resistance and the practical difficulties in delivering health care to rural communities, most of those at risk of malaria have not had such access [3]. However, with the development of artemisinin derivative combination therapy (ACT) and international financial support to allow inexpensive or free distribution to those in need, hope of controlling malaria has been rekindled [3]. A major obstacle to malaria control, which has received woefully insignificant attention in the past, is the poor quality of antimalarial medicines available in much of the tropics. The available data strongly suggest that antimalarials have been particularly targeted by counterfeiters, who have deliberately and fraudulently produced copies for profit, usually containing no active ingredients and therefore lacking antimalarial activity [4].

Artesunate is an antimalarial artemisinin derivative, developed in the People's Republic of China (China), vital for malaria treatment [5]. It is widely used in South East (SE) Asia and increasingly in Africa for the treatment of Plasmodium falciparum malaria [3]. In SE and East Asia there are at least 16 manufacturers of artemisinin and its derivatives [6] and millions of tablets are produced each year both for consumption in Asia and export to Africa. Artesunate is a crucial component of artemisinin derivative-based combination therapy, but is often used inappropriately as monotherapy outside of national malaria programmes.

**Figure 1:**
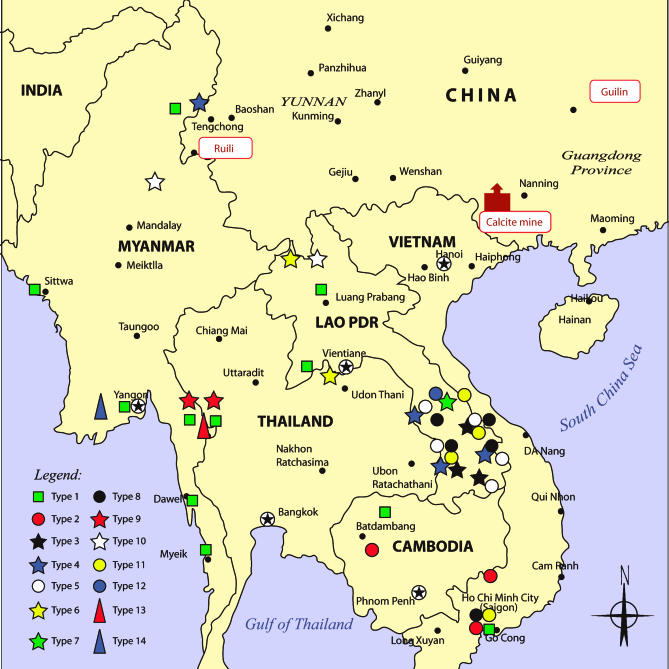
Map of the Distribution of Fake Artesunate, Collected by Wellcome Trust-University of Oxford SE Asian Tropical Medicine Research Programme and Collaborators, in Relation to Packaging Type Map drawn by Mr. Chongkham Phonekeo.

**Figure 2:**
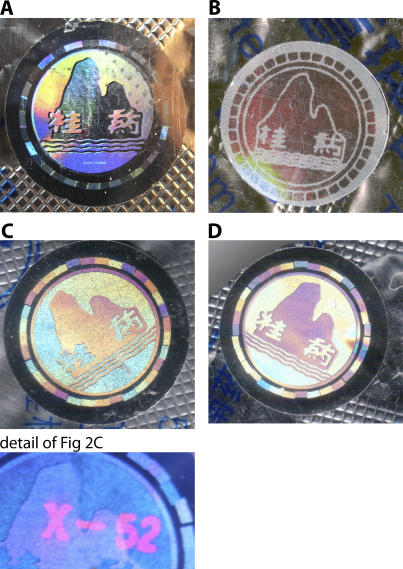
Examples of Genuine and Counterfeit Holograms (A) Genuine Guilin Pharmaceutical artesunate blister pack hologram. (B) A type 2 sticker copy. (C) A type 10 hologram copy with the fake ‘X-52′ stamp as seen under UV light. (D) Type 15 hologram copy from the seized counterfeit artesunate from the China/Myanmar border. For a full description of the 16 types of fake packaging see Figure S1 (updated from [10]). Types 15 and 16 have been only recently described and were not included in the pre-seizure analysis presented here. Photographs by Paul Newton.

Since 1998 an epidemic of multiple types of counterfeit artesunate tablets, both primitive and highly sophisticated copies, have affected patients with malaria in mainland SE Asia (Figures 1, 2, and S1). Ad hoc surveys since 2000 in Burma (Myanmar), the Thai/Myanmar border, the Lao People's Democratic Republic (Lao PDR, Laos), Cambodia, and Vietnam, the most malarious countries in mainland SE Asia, suggested that 33%–53% of bought artesunate was counterfeit, containing either no or subtherapeutic quantities of artesunate (Figure 1) [7–13]. In Thailand, which has only a small number of malaria cases, the threat of counterfeit artesunate has been largely averted: such counterfeits have only been described from the central Thai/Myanmar border area around the Western border town of Mae Sot. Counterfeit artesunate has been recently described from southern China [14]. In Asia the product of one major producer of artesunate, Guilin Pharmaceutical (Guilin, Guangxi autonomous region, China), has been exclusively targeted. The recent description of counterfeit artemisinin derivatives in four sub-Saharan African countries is of enormous public health concern [10,15–17].

Counterfeit artesunate containing subtherapeutic quantities of artesunate has recently been described in Asia [10]. Such counterfeits, which may foil simple screening tests, will engender the selection and spread of artemisinin resistant falciparum parasites, which would be disastrous for malaria control in Asia and thereafter in Africa.

Despite the difficulties of demonstrating in rural Asia that patients die of malaria because of fake drugs, such mortality has been reported [10]. Malaria remains a public health problem in the countries affected by fake artesunate—with estimates of over 2 million cases/year and over 10,000 deaths/year in the Western Pacific Region of the World Health Organization (2001 data [18]). With an estimated prevalence of counterfeit artesunate of 33%–53% in mainland SE Asia and a high proportion of patients obtaining antimalarials outside the public health provision of ACTs, the health impact on malaria morbidity and mortality must be considerable—but extremely difficult to quantify.

Since the first description of fake artesunate in 2000, there has been little action in comparison to its public health consequences, apart from surveys [8–13], warnings and educational films [10, 19] with some strengthening of drug regulatory authority (DRA) and malaria programme capacity. With little progress and worsening contamination of the supply of antimalarials in the Greater Mekong Sub-Region, a confidential meeting was held in Manila in May 2005 at the WHO Regional Office for the Western Pacific (WPRO). The meeting brought together WHO officials, physicians, pharmacists, and scientists working in the region with the International Criminal Police Organization (INTERPOL) to discuss what could be done. It was decided that a joint effort be made to investigate where the counterfeits were being manufactured and develop an intelligence document that could be presented to concerned governments with a request that measures be taken to stop the lethal manufacture and trade in counterfeit artesunate.

In the hope that forensic analysis of genuine and counterfeit tablets would provide clues as to the origin of the counterfeits, samples were subjected to high performance liquid chromatography (HPLC), organic mass spectrometry, X-ray diffraction, stable isotope ratio mass spectrometry, gas-chromatographic ‘head space' analysis of the gases surrounding tablets in blister packs, pollen analysis (palynology), and detailed packaging inspection. Further meetings were held in Oxford, UK, and Manila to review the evidence under the umbrella of the Jupiter Operation INTERPOL anti-counterfeiting taskforce chaired by JN. This paper presents the findings of this group relevant to public health.

## Methods

### Samples

391 samples of genuine and counterfeit artesunate collected in Vietnam (75), Cambodia (48), Lao PDR (115), Myanmar (Burma) (137) and the Thai/Myanmar border (16), during 1999–2006 by the Wellcome Trust-Oxford SE Asian Tropical Medicine Research Programme, underwent Fast Red TR Dye testing [20] and/or HPLC and mass spectrometry and analysis of packaging. These samples were collected and analysed as a part of formal surveys using convenience sampling [8,9,11] and random sampling (in Laos only, unpublished data) and ad hoc at the request of individuals and non-governmental organisations in the region.

A subset of 27 fakes from this collection and five genuine packets, along with four fake (collected in Cambodia) and two genuine samples from the United States Pharmacopeia (USP) Drug Quality and Information Program, representing a sample of the oral artesunate labelled as made by Guilin Pharmaceutical available in 2005, also underwent detailed forensic chemical and botanical examination as part of the Jupiter operation. Results, some of which were partially described before [8–11,13,21–24], for the 391 samples and for the 49 samples, including 11 seizure samples (see Action and Examination of Seizures), subjected to detailed analysis are presented and compared here.

Five samples seized by the subsequent Ministry of Public Security (MPS) investigation in China were also analysed. All laboratories performed the analyses blinded to the results from other laboratories.

### Physical Appearance

The physical appearance and text on packets, leaflet inserts, and blister packs were examined and compared with known genuine samples. Packets, leaflets, and blister packs were examined with a ×6 hand lens, ×100 stereo microscope, and a hand-held UV (375 nm) light source and then electronically scanned. Batch numbers, dates of expiration and manufacture, the colour, clarity, and text of printing on the blister pack, packet, and leaflet (when present) were documented. Guilin Pharmaceutical kindly informed us which batch numbers were genuine for samples labelled as manufactured by this company.

### Chemical Investigations

Tablets were processed and analyzed for artesunate according to a modified HPLC method [25] (Protocol S1). Samples were screened by two different direct ionization mass spectrometry methods: Direct Analysis in real Time (DART) [21] and Desorption Electrospray Ionization (DESI) [22]. For validation purposes, DESI was performed in two modes: reagentless DESI and reactive DESI. Three mass spectra were thus provided for each sample (Protocol S2). A small portion of each sample was analysed by X-ray diffractometry (XRD; X'Pert Pro X-ray diffractometer, Philips, Almelo) and isotope ratio analysis to determine the mineral composition (Protocol S3) [26,27].

### Biological Investigations

Tablet samples were processed (Protocol S4) and slides examined at a magnification of ×125–250 for about 1 h per slide. Any suspected pollen grains were examined and identified at a magnification of ×500–1,000 by comparison with reference material and compared with the known distribution of the taxa found (GNS Science reference modern spore and pollen collection [28–32]). These results should be interpreted with caution as the spore and pollen content could be indicative of either the place of manufacture, the source of the individual ingredients, or both, and be influenced by their wide wind dispersal and seasonality. Invertebrate remains and other organic materials embedded in the tablets were also identified.

### Intelligence

Associations between location and chemical, biological, and packaging features were examined and geographical distributions mapped. Statistical analysis was performed using Stata v8. These results were presented to the Ministry of Public Security, People's Republic of China, who carried out a confidential criminal investigation to track the traders and factories responsible for this illegal activity in collaboration with the Government of Myanmar.

## Results

### Physical Appearance

A total of 14 different fake hologram types were identified, between 1999 and May 2006, ranging from crude stickers to highly sophisticated copies, complete with the legend ‘Guilin Pharma' in microscopic font below the illustrated river and the Chinese registration code ‘X-52′ visible under UV light stamped on the hologram (Figure 2). A further two hologram types were identified between June 2006 and February 2007, after the presentation of the evidence to the Chinese authorities (Figure S1). The outer rings of all counterfeit holograms were composed of misaligned blocks. Many other small differences between the counterfeit and genuine were noted. There were printing errors in boxes, with misalignment of letters and words, differences in font, and spelling mistakes, such as the spelling of Tablet as ‘Tabtle' on type 4 blister packs. The text on the blister pack was printed with offset on genuine samples but by serigraphy (silkscreen) on all seven fake samples examined (types 1, 4, 6, 9, and 10), resulting in thicker ink deposit and poorly defined text. All genuine and counterfeit boxes had a bar code printed on the reverse (6920108100058), which was successfully decoded with a handheld bar code scanner for seven fake samples examined. Until 2005 the golden foil and Myanmar Registration Number, characteristic of Guilin Pharmaceutical artesunate exported to Myanmar (Burma), had not been used in the fakes—but samples with both these features appeared in 2006 (type 14).

Hologram inspection demonstrated that the central image of the genuine hologram was made in ‘2D–3D' style from flat graphic line artwork [13]. Fake stickers such as type 2 were made from diffractive ‘rainbow' foil deeply etched to form an image. The type 3 fake hologram is a simple 2D origination comprised of two exposures of diffractive colour, one of which is much brighter than the other. The type 4 fake hologram is very similar in appearance to the genuine hologram, despite being manufactured using different techniques, comprised of a 2D image in two contrasting colours. Unlike the genuine hologram, both diffractive colours are made using the McGrew method (with diffused object beams, rather than undiffused gratings) and thus have higher brightness than the genuine hologram. As in the genuine hologram, the kinetic feature of the outer ring is formed of sections in three different diffractive colours (two of which are the same colours as the main image). In type 4 these colours are repeated to provide an apparent animation when the hologram is tilted vertically, rather than right-to-left as in the genuine hologram [13].

### Chemical Composition

HPLC, Fast Red Dye testing [20], and/or mass spectrometry confirmed that all specimens thought to be counterfeit (195/391, 49.9%) on the basis of packaging contained no or small quantities of artesunate (up to 12 mg per tablet as opposed to ∼ 50 mg per genuine tablet). One sample from Cambodia had apparently genuine packaging but contained only 21 mg artesunate and was therefore either substandard or had deteriorated in transit and/or storage [13]. Of 321 samples labelled as made by Guilin Pharmaceutical, 195 (61%) were counterfeit. Of 51 artesunate counterfeits that underwent mass spectrometry, a wide variety of unexpected ‘wrong' pharmaceuticals were found, including paracetamol (*n* = 24), sulphadoxine (*n* = 18), pyrimethamine (*n* = 11), dimethylfumarate (*n* = 17), erythromycin (*n* = 8), erucamide (*n* = 5), safrole (*n* = 6), artemisinin (*n* = 10) metamizole (*n* = 10), 2-mercaptobenzothiazole (*n* = 5), chloramphenicol (*n* = 4), metronidazole (*n* = 2) and chloroquine (*n* = 2). In addition, four samples contained subtherapeutic amounts of artesunate within fake packaging; (Tables S1 and S2) [7, 21]. A wide variety of different erythromycins (erythromcin A–F and anhydroerythromycin A, 20–191 mg/g tablet) have also been described from fake artesunate with considerable inter-tablet variation in those present [13]. In addition, three samples collected in the USP Drug Quality and Information Program in the Jupiter subset from Cambodia (types 3 and 10) contained sulfamethoxazole (62, 66, and 0.06 mg/tablet) and XRD demonstrated the presence of decanoic acid and metamizole in a type 10 sample. Headspace analysis demonstrated the presence of ethanol and chloroform in the gas surrounding apparently genuine Guilin Pharmaceutical artesunate tablets from four blister packs (chloroform 1–2 μg/g). Chloroform and benzene were found in the headspace of a type 4 blister pack (chloroform 1 μg/g, benzene 1–14 μg/g) and chloroform in a type 1 blister pack from the Thai/Myanmar border.

**Figure 3:**
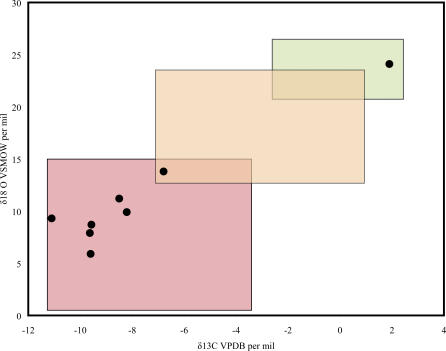
Plot of δ^13^C and δ^18^O Values, in ‰, of Carbonate in Tablets Also plotted are approximate fields for values expected from calcite in different geological environments; igneous (red), metamorphosed (pink), and unaltered limestone (green). Sample 05/17 is represented by the top right data point.

XRD demonstrated great variability in tablet mineralogy. Talc (hydrated magnesium silicate), calcite (calcium carbonate), and starch were the main excipients detected. Maize starch is the main excipient in the genuine Guilin Pharmaceutical product. Trace amounts of aragonite were present in two calcite-dominated fake tablets and traces of chlorite (iron-magnesium hydrosilicate) and quartz (silicon dioxide) were found in a further two (Table S1). Stable isotope analysis of the calcite fraction gave δ^18^O and δ^13^C values between −11‰ to +2‰ and +2‰ to +25‰, respectively (where ‰ indicates parts per thousand) (Figure 3). Given the similar isotope signatures the calcite in all (types 2, 4, 5, 8, and 11), but one sample, appears to come from the same source and is either hydrothermal or medical in origin. The one exception is sample 05/17 (type 12) from southern Laos (Table S1) which, if the calcite is assumed to have been formed by natural geological processes, is typical of marine limestone (Figures 1 and 3).

### Biological Composition

**Figure 4:**
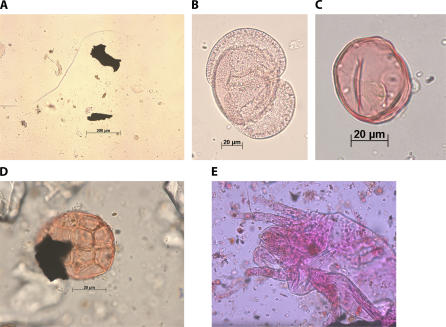
Photomicrographs of Material Found in Fake Artesunate Tablets (A) Debris, including charcoal fragments. (B) *Pinus* pollen grain. (C) *Juglans* pollen grain. (D) *Acacia* pollen grain with charcoal deposit. (E) *Dermatophagoides* mite nymph. The maximum distance across the abdomen is 116 μm. Photographs by Dallas Mildenhall.

Most of the spores and pollen recovered have a distinct northern Asian temperate signature with evidence for plants such as firs, pines, cypresses, sycamores, alders, wormwood, willows, elms, wattles, and numerous fern spores present (Figure 4; Table S1). The estimated origin of the samples includes northernmost Vietnam and southern China, and certainly northern SE Asia near the mountains bordering Myanmar (Burma) through to northern Vietnam. Some of the plant remains reflected the original plant source of the starch. The presence of Juglandaceae pollen (*Juglans* [walnut], *Carya* [wing nut], and *Pterocarya* [pecan nut or hickory]) in some samples suggests manufacture in southern China. This family is common throughout SE Asia but, except for the north east coastal plains of China, dies out in southern China north of the borders with Myanmar (Burma), Vietnam, and Laos [33]. Three of the 11 samples containing calcite have little or no spore/pollen contamination suggesting that the contamination does not come from the calcite or its source. In terms of the different types of fake hologram/sticker, type 2 appears different from the others with no smooth trilete fern spores; type 1 contained little material but appears similar to type 3; types 10, 11, and 12 had similar pollen signatures. Types 5 and 8 did not contain anything to enable comparisons and types 4, 6, 7, 9, 13, and 14 were not examined.

Although we do not know whether the date of manufacture stamped on the fakes is the actual date they were made, in general the flowering times of the plants whose pollen occur in the samples agree with the date stamped on the blister packs. The two exceptions are samples 2267 and 2/12051 with a date stamp of September 2003. In addition to plant remains, five samples contained invertebrates, including a *Dermatophagoides* mite nymph (from type 11, Vietnam, Figure 4E), which is commonly found in house dust and has a global distribution. All fake artesunate tablets contained charcoal fragments, presumably from vehicle exhausts and fires, broken up by the processing for pollen. In some samples, charcoal was so abundant that it suggested the source was an area suffering severe air pollution.

### Interpretation of the Evidence

The pooling of chemical, biological, and packaging evidence suggests a wide diversity of fake artesunate in SE Asia with strikingly different fake holograms and chemical signatures. However, palynological evidence suggests that all were produced in relatively temperate areas on the China/SE Asia borders. Inspection of maps of the geographical distribution of fake artesunate suggests that there may be two different origins of counterfeit artesunate, with similarities between the samples in Myanmar (Burma), the Thai/Myanmar border and northern Laos in a westerly group and similarities between those recovered in southern Laos, Vietnam, and Cambodia in an easterly group (Figure 1). Of the 319 samples labelled as made by Guilin Pharmaceutical, with a detailed location recorded, there were a significantly higher proportion of counterfeit drugs in the east than in the west (41% (62/151) versus 79% (133/168), *p* < 0. 001). In the west 73% (37/51) of batch numbers used were invalid whereas in the east only 1.5% (2/128) were invalid (χ^2^ = 107.8, *p* < 0.001). Classifying fake ‘holograms' as either stickers or holograms, fake holograms were affixed to 73% (19/26) of fake blister packs in the west but only 11% (13/120) in the east (χ^2^ = 48.4, *p* < 0.001). Fakes with stickers were only found in Vietnam, Cambodia, and northern and southern Laos whilst those with holograms were only found in Myanmar (Burma), the Thai/Myanmar border, and both northern and southern Laos. Samples collected in Cambodia and Vietnam bore fake stickers and none with fake holograms was found in Vietnam. The fake holograms all have mis-registration of the outer ring and although mis-registrations differ between all types, they have been made in the same way, suggesting the use of similar technology and machines (D. Pizzanelli personal communication). Types 2, 3, 5, 7, 11, and 12 were only found in the east whilst 6, 9, and 10 were only found in the west. 82% of type 1, 17% of type 4, and 25% of type 8 were found in the west. With the exception of type 4, all those containing calcite bore stickers and not fake holograms. However, the pollen signatures of types 3, 11, and 12 (easterly) and 9 and 10 (westerly) were similar, suggesting that the manufacturing sites of these two groups may be in the same areas of southern China, but that they are separate operations with different distribution networks. The upper case font error for ‘Mfg' and ‘Exp', found only on blister packs of types 8, 11, and 12, suggests a link between the producers of these three types. The occurrence of a type 10 blister pack in a type 4 box and a type 13 blister pack in a type 9 box suggests links between the producers and/or distributors of these fakes. These links are consistent with the ‘easterly' and ‘westerly' distribution hypothesis. The existence of two main sources is also suggested by the chemical analysis. Metronidazole and artesunate were only found in westerly group samples and sulphadoxine, pyrimethamine, erythromycin, and erucamide were found only in the easterly group samples. Dimethylfumarate (88%), 2-mercaptobenzothiazole (80%), metamizole (70%), safrole (67%), paracetamol (67%), and artemisinin (60%), were found predominantly in the east. Samples containing chloramphenicol and chloroquine were found in equal numbers in each geographical group.

The presence of numerous wrong active pharmaceutical ingredients in counterfeits may provide clues as to their origin(s). The observation that counterfeit artesunate (type 14) actually contained artesunate, but only on the surface, suggests that the counterfeit tablets may have been contaminated with the genuine ingredient in a tablet press left over from the manufacture of artesunate at an unknown location [22]. The presence of sulphadoxine and pyrimethamine suggests that the factory had been making or had access to sulphadoxine–pyrimethamine, an antimalarial no longer efficacious in SE Asia. The discovery of safrole is of considerable concern and interest as it is a precursor in the synthesis of methylenedioxymethamphetamine (MDMA or ecstasy) and is a banned ‘Table 1' chemical in the United Nations Convention Against Illicit Traffic in Narcotic Drugs and Psychotropic Substances [34]. Concerned that the counterfeits may have been coming from the illicit methamphetamine factories in Asia, we attempted to detect, but failed to find, methamphetamine or MDMA in or on the counterfeits.

All the counterfeits analysed from Vietnam contain calcite. The C and O stable isotope ratios of the calcite are consistent with a geologically high temperature intrusive or volcanic origin and not with a marine or terrestrial limestone or tap water precipitation origin (except one, type 12). The presence of a small amount of aragonite could indicate marine limestone origin but heavy industrial crushing will convert non-marine calcite to aragonite, which is also found in open fissures in volcanic rocks. High temperature hydrothermal calcite is mined in an area west of Nanning (Guangxi autonomous region, China) close to the Vietnam border (Figure 1) [35] and the calcite used in types 2, 4, 5, 8, and 11 may have come from these mines.

**Figure 5:**
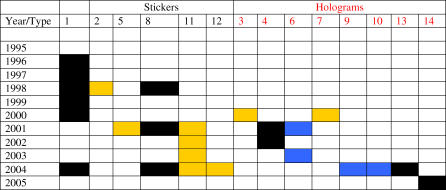
Relationship between the Stated Year of Manufacture of Fake Artesunate for Those Bearing Fake Stickers and Fake Holograms in Order of Discovery between 2000 and 2006 Black, found in east and west; yellow, found in easterly distribution only; and blue, found in westerly distribution only.

The combination of excipient composition and palynology therefore suggested that at least some of the counterfeit artesunate were coming from southern China, close to the border with Vietnam, Laos, and Myanmar (Burma). Furthermore, the evidence suggests that there are at least two origins of this ‘product' with westerly origins via Myanmar (Burma) and easterly origins via Vietnam trade routes with Laos in the middle as an area of overlap afflicted by types of both groups. In Figure 5 the types of fake hologram/sticker are plotted versus the stated date of manufacture in the order they were discovered in SE Asia. The first fakes without stickers or holograms (type 1) were labelled as made in 1996, whilst the first with fake stickers appearing in 1998 and the first with fake holograms in 2000. There has been a trend of increased sophistication of the fake holograms but not of the fake stickers. Some confidential security features present on the genuine were not copied on the counterfeits perhaps because of the cost and difficulty of doing so or because the counterfeiters were ignorant of their presence.

### Action and Examination of Seizures

The evidence described here was presented, by the Secretary General of INTERPOL (Mr Ronald K Noble) to the Senior Representative of the Assistant Minister of Public Security (MPS, Mr Zheng Shaodong), in March 2006 in Beijing. The following issues were discussed in relation to the evidence that the counterfeits were being produced in southern China and distributed in large amounts into adjacent malarious countries: (1) the enormous clinical importance of artemisinin derivatives, which had been discovered and developed in China, in the global treatment and control of malaria; (2) the public health impact of counterfeits in Asian and potentially African countries; and (3) the economic consequences for Chinese producers, such as Guilin Pharmaceutical, the main producer of genuine artesunate—a WHO prequalified product.

In response the MPS carried out a criminal investigation and arrested a native of Yunnan Province who, it was alleged, had bought 240,000 blister packs of counterfeit artesunate from a native of Guangdong Province, China who was also arrested. 160,000 of these blister packs were alleged to have been sold to a non-Chinese national on the Yunnan and Myanmar (Burma) border, followed by a further 56,000 blister packs at another border crossing on the Yunnan/Myanmar border near Ruili whilst 24,000 blister packs were seized by the MPS. The producer of counterfeits is thought to be a Chinese national and that his factory is in Guangdong province. Those arrested await trial. During the approximate period of this trade Guilin Pharmaceutical exported 272,000 blister packs to Myanmar (Burma) and Thailand. Therefore, of the visible trade in 512,000 blister packs of artesunate, 47% was in fake artesunate, consistent with previous estimates of the proportion of fake artesunate in mainland SE Asian shops.

Five samples of the seized counterfeit artesunate were compared with six contemporary genuine samples from Guilin Pharmaceutical. Of the counterfeit artesunate holograms previously described from SE Asia those in this seizure most closely resembled type 10 (Figures 2 and S1) but did not have the ‘X-52′ symbol visible on the hologram under UV light on type 10. We have therefore described this as type 15. Presumably this hologram has come from the same source as type 10, but the X-52, which is stamped on the hologram surface, was omitted. The pattern of ring blocks in the outer circle in type 15 appears identical to that of type 10, which has been described from northern Myanmar (Burma) and northern Laos. The text ‘12 Tabs' printed on the end tab of the type 15 packet, had a terminal ‘.' (i.e., ‘12 Tabs.') on the genuine packets but no terminal ‘.' (i.e., ‘12 Tabs') on the counterfeits.

The median (range) concentration of artesunate in contemporary genuine tablets analysed by HPLC was 48.4 (46.8–48.6) mg/tablet. The five counterfeit samples contained artemisinin, and not artesunate, with a median (range) concentration of 14.6 (14.1–15.1) mg (Table S1). As artemisinin is considerably less active than artesunate, this very small dose would have served only to encourage resistance, and would not have cured malaria. No other active pharmaceutical ingredients or calcite were detected in the counterfeit tablets by mass spectrometry and XRD, respectively. Palynology gave no evidence that the source of the tablets is outside of southern China, but the types of pollen and spores recovered are wide-spread types and do not exclusively include southern China as the source area.

The seized samples were most clearly related to type 10 (and hence also type 4) in terms of packaging and were related to the more sophisticated westerly counterfeits found in Myanmar (Burma), Thai/Myanmar border, and Laos bearing sophisticated holograms and without the plethora of wrong active ingredients (other than artemisinin). The absence of calcite in the seized counterfeits also supports the hypothesis that these samples were produced by the group of criminals involved in the putative western arm of the trade in counterfeit artesunate distributing their product into Myanmar (Burma), down to the Thai/Burma border and across into Laos through northern Myanmar (Burma) or southern China. This leaves open the possibility that there is a second trade route, which has not yet been disrupted, involving a different group of criminals distributing and/or manufacturing fake artesunate, with poor quality stickers and a variety of wrong ingredients with calcite, into Vietnam, Cambodia, and southern Laos. If this hypothesis is correct future examination of the prevalence of different types would be expected to demonstrate a reduction in the westerly distribution, but not that fed by the easterly trade route. We suggest that the criminals responsible for the easterly trade route have also manufactured or have links to manufacturers of sulphadoxine–pyrimethamine, erythromycin, and possibly to MDMA.

## Discussion

The Jupiter group of scientists, criminal analysts, and health workers was formed in an attempt to provide information that could be used to control the epidemic of counterfeit artesunate in SE Asia which had not been curbed by pre-existing collaborations and institutions. The forensic evidence suggested that at least some of the counterfeit artesunate were coming from southern China, and that there are at least two origins of this ‘product'—with westerly and easterly origins and Laos in the middle as an area of overlap afflicted by types of both groups. This evidence precipitated a police investigation by the Chinese Government. Shortcomings of the evidence include that the artesunate collections were ad hoc and not collected with a legal chain of evidence and only a small subset of the collection could be analysed due to financial constraints. However, the samples analysed did point to the apparent source of the fakes and it is unlikely that a larger sample size would have changed the outcome.

Further evidence was also obtained of the potential harm of these criminal products, both to individual patients and public health. A wide diversity of potentially dangerous wrong active ingredients has been described from fake artesunate tablets. The presence of unexpected pharmaceuticals, such as chloramphenicol and metamizole, may lead to inexplicable clinical syndromes such as bone marrow failure. Metamizole is banned in the European Union for this reason. Benzene and safrole are Class 1 and 2B carcinogens, respectively [36]. In addition, the use of ‘artesunate' containing subtherapeutic quantities of artesunate, artemisinin, and sulphonamides greatly increases the risk of the emergence and spread of drug-resistant malaria parasites. A wide variety of bacterial illness can be confused clinically with malaria and the inadvertent use of subtherapeutic concentrations of antibiotics such as erythromycin, chloramphenicol, and metronidazole for such bacterial infections could also engender the spread of antibiotic resistant bacteria.

Although there is a relationship between packaging and chemistry this is not absolute, presumably in part because the criminals change their recipes depending on what chemicals they have available, out of synchrony with changes in packaging. As there are few distinguishing external features it is likely that type 1 has multiple sources and that we have wrongly ‘lumped' these together as is suggested by their variable chemical composition [13]. Why so many different pharmaceuticals are present is unclear but may simply be the powders available to the counterfeiters. However, some wrong ingredients, such as chloroquine (which is not efficacious against falciparum malaria in Asia), may have been added to give a bitter taste as there is a tradition in Asia that antimalarials should be bitter. Furthermore, small quantities of subtherapeutic artesunate may have been added to fool the Fast Red Test for the rapid determination of authenticity [20].

Forensic palynology has been used to determine the location of murders [37–39] and the provenance of illicit drugs [40] but, to our knowledge, it has not been used to determine the location of the manufacture of counterfeit pharmaceuticals or the origin of their ingredients before. However, the results should be treated with some caution in that the spore and pollen content could represent the place of manufacture, the source of the individual ingredients, or both, and some pollen types can travel large distances on the wind. Most pollen identified were from plants whose natural distributions are northern temperate or sub-tropical montane and not tropical types, consistent with a presumed northernmost SE Asia source. Pollen from *Artemisia* was found in two of six genuine and three of 18 counterfeit samples in the Jupiter subset. It is possible that this pollen was derived from the source plant for artemisinin, Artemisia annua, but the pollen does not allow speciation. *Isoetes* species spores (found in type 11) are endangered aquatic plants found in east and SE Asia [41]. The presence of spores and pollen in the tablets are more a reflection of source than flowering times as these palynomorphs have the ability to survive after dispersal for over a year and can be picked up during the processing of the tablets at any time of year. However, many of the tablets seem to have been processed during or close to the flowering season of the plants identified. Those containing sparse spores and pollen are not necessarily from different sources, just evidence of a cleaner operation.

Outside of the large pharmaceutical companies there has been little international collaboration between police and those concerned with the public health problem of counterfeit life-saving drugs. Large pharmaceutical companies are active in investigating and facilitating prosecutions, but these actions are rarely reported publicly and smaller companies in the tropics do not have the resources to act. Such criminal investigation and legal action is important in disrupting and inhibiting the trade in counterfeit medicines. For example, cooperation between the Belgian and Chinese authorities resulted in the interception of 57,000 packs of counterfeit halofantrine capsules en route from China to Nigeria and arrests [42]. However, such forceful action needs to be part of multiple linked tactics, including increasing the accessibility of patients with malaria to inexpensive antimalarial drugs, facilitating inspection by DRAs (30% of which have no capacity or barely function [9]), and policing of the drug supply.

Our investigation was able to make evidence-based suggestions as to where at least some of the fake artesunate was being manufactured. The involvement of INTERPOL was crucial, acting as a bridge between the health sector (including WHO and the physicians and scientists) and national police agencies to act as a catalyst for action. Indeed, once presented with this evidence the Chinese police authorities acted quickly to stop production and dissemination. We suggest that this model of collaboration between criminal investigators, forensic scientists, physicians, and pharmacists is a useful approach in further combating this scourge. The counterfeiters of anti-infectives have killed patients with impunity. However, the use of new criminal forensic tools may facilitate the detection of counterfeits and greatly increase the risks to counterfeiters of being caught. However, if this collaborative approach is to be used to counter other counterfeit drug public health problems, further investment in forensic analysis and the building of sustainable links between relevant organisations will be required. Police investigations of these trans-national crimes without specialised evidence are unlikely to be successful. Forensic evidence would allow overstretched police forces to focus on these objective leads. There are very few laboratories (only one in Asia/Pacific) with the appropriate reference collections and ability to analyse pollen assemblages in tablets and the work is very labour intensive and specialised and therefore expensive. Similarly, there are very few (less than ten) public laboratories or research groups with the adequate tools and human resources to perform detailed forensic chemistry of fakes and the equipment required is expensive. We were fortunate in that diverse international and national bodies were able to provide their time and skill to try to solve a single important problem.

The evidence available suggests that poor drug quality is a major problem disabling public health in Africa [4,10,16,43]. If a collaborative forensic approach is to succeed, DRAs and their international collaborations will need to be strengthened and regional laboratories able to provide actionable evidence will be required.

## Supporting Information

Figure S1 Warning Sheet, Describing 16 Different Types of Counterfeit Artesunate (1.4 MB PDF)

Protocol S1 High-Performance Liquid Chromatography (20 KB DOC)

Protocol S2 Mass Spectrometric Analysis (38 KB DOC)

Protocol S3 X-Ray Diffractometry (20 KB DOC)

Protocol S4 Botanical Analysis (22 KB DOC)

Table S1 Summary of Packaging, Chemical, and Botanical Analysis of ‘Artesunate' Samples Samples judged to be counterfeit on the basis of packaging characteristics, blinded to the results of chemical analysis, are given in bold.
(146 KB DOC)

Table S2 Description of Different Types of Counterfeit Artesunate According to Counterfeit Hologram Type for Whole Dataset Stickers—plain text; holograms—bold text. Includes data published in Hall et al. (2006) [13], Fernández et al. (2006) [21], Nyadong et al. (2007) [22], and Newton et al. (2006) [10].
(86 KB DOC)

## Abbreviations

DRA: drug regulatory authority
HPLC: high performance liquid chromatography
INTERPOL: International Criminal Police Organization
MDMA: methylenedioxymethamphetamine or ecstasy
MPS: Ministry of Public Security
SE Asia: South East Asia
WPRO: Western Pacific Regional Office (of WHO)
XRD: X-ray diffractometry
