# Supplementary material for: A Collaborative Epidemiological Investigation into the Criminal Fake Artesunate Trade in South East Asia
Source: PLoS Med. 2008 Feb 12;5(2):e32. doi: 10.1371/journal.pmed.0050032 (PMC2235893; doi:10.1371/journal.pmed.0050032)
Supplement: Supplementary file 2 [file pmed.0050032.sd001.doc]

**Protocol S1.**  High Performance Liquid Chromatography

Tablets were processed and analyzed for artesunate according to a modified High Performance Liquid Chromatography (HPLC) method [25]. A 150 x 4.6 mm octadecylsilica column and a mobile phase consisting of 45% acetonitrile and 55% 0.05 M perchlorate buffer (pH 2.5) effectively separated artemisinin, paracetamol, and artesunate. For quantitative paracetamol analysis, the mobile phase was changed to 10% acetonitrile and 90% perchlorate buffer. Components were detected using a diode array detector with wavelengths set at 220 and 254 nm and UV spectra of samples were compared with artesunate, artemisinin and paracetamol reference standards for validation.
