# Supplementary material for: A Collaborative Epidemiological Investigation into the Criminal Fake Artesunate Trade in South East Asia
Source: PLoS Med. 2008 Feb 12;5(2):e32. doi: 10.1371/journal.pmed.0050032 (PMC2235893; doi:10.1371/journal.pmed.0050032)
Supplement: Supplementary file 3 [file pmed.0050032.sd002.doc]

**Protocol S2. Mass Spectrometric Analysis**

Mass Spectrometry (MS) experiments were performed by two complementary methods:

1. Direct Analysis in Real Time (DART) MS.
2. Desorption Electrospray Ionization (DESI) MS.

- Reagentless-DESI MS
- Reactive-DESI MS

A more detailed general description of the DART and DESI techniques can be found in the following references:

1.	Cody R, Laramee J, Durst H (2005). Versatile new ion source for the analysis of materials in open air under ambient conditions. *Anal Chem* 77: 2297-2302.

2.	Takats Z, Wiseman JM, Cooks RG (2005). Ambient mass spectrometry using desorption electrospray ionization (DESI): instrumentation, mechanisms and applications in forensics, chemistry, and biology. *J Mass Spectrom* 40: 1261-1275.

**DART-MS analysis**

Direct Analysis in Real Time (DART) is a high-throughput ionization method for mass spectrometry (MS). In DART MS, a special type of ion source is used. This ion source produces a heated stream of protonated reactant ions, which are directed towards the tablet under investigation. The stream desorbs chemical species from the tablets, simultaneously ionizing them. These ions are generated in the open air between the ion source and the mass spectrometer inlet, and sampled by a small orifice to generate a mass spectrum. If a high resolution time-of-flight (TOF) mass spectrometer is used, accurate mass experiments are possible.

*Experimental:* DART measurements were performed with a JMS-100TLC (AccuTOF™) TOF mass spectrometer (JEOL, Peabody MA, USA,) equipped with a DART ion source. Experiments were performed in positive-ion mode, at a temperature of 250°C. Samples were manually held with a pair of clean metal tweezers in front of the DART ion source for approximately 20 s. The settings for the mass spectrometer were those recommended by the manufacturer. Mass spectra were acquired in the 100 to 1000 Da range.

For the purpose of identifying unknowns in counterfeit tablets, the spectrometers’ mass drift was corrected using neat poly(ethylene glycol) (PEG, average molecular weight 600, Sigma Aldrich, St. Louis MO, USA). DART mass spectra were exported into MS Excel, and searched, via a system of macros, against an in-house library of exact masses for protonated molecules derived from the Model List of Essential Drugs published by the WHO and the list compiled by Gergov et al. J. Chromatogr. B, 2003, 795, 41-53. A match was considered positive if the difference between the experimental accurate monoisotopic mass and the theoretical mass values was less than 5 mmu, and the relative intensity equal or larger than 5 %.

**DESI-MS analysis**

In DESI MS analysis, a high-speed electrically-charged liquid spray is directed at the tablet under inspection. This tablet is kept at atmospheric pressure, outside of the mass spectrometer. The DESI spray progressively dissolves material from the tablet, giving a qualitative depth profile. The charged droplets containing tablet material are sampled downstream by a mass spectrometer, providing a spectrum of the sample components. No tablet dissolution is required making this technique well-suited for screening large number of samples. Several points on the tablet surface and interior were sampled during DESI analysis to obtain an accurate picture of the components present [23].

*Experimental:* A homebuilt DESI ion source was used in all experiments [22]. Experiments were performed on an LCQ DECA XP+ ion trap mass spectrometer (Thermo Finnigan, San Jose CA, USA). Reagentless DESI was performed in positive ion mode by spraying a 75:25 CH_3_CN:H_2_O solution onto the tablets at a flow rate of 5 μL/min. HPLC grade acetonitrile (Fisher, Hampton NH) and ultrapure water (18.2 MΩ cm^-1^) were used in all DESI experiments. Reagentless DESI allows detection of species with larger molecular weights than DART MS. Data was acquired via the Xcalibur software interface for a total acquisition time of 4 minutes. The following scan method was used:

| ***Scan time*** | ***Scan type*** | ***Comments*** |
| --- | --- | --- |
| 0-0.2 min | High repetition rate scan in 100-1000 *m/z* range (TurboScan) | Superficial species on the tablet outermost layers are monitored. |
| 0.2-0.7 min | Full scan in 400-410 *m/z* range at normal rate. | High sensitivity scan for artesunate [M+Na]^+^ (*m/z*=407) in inner tablet layers. |
| 0.7-1.2 min | Full scan in 100-1000 *m/z* range at normal rate | Scan for artesunate [M+Na]^+^, [2M+Na]^+^, and unknown species in inner tablet layers. |
| 1.2-4 min | Data dependent MS^2^ scan in 100-1000 *m/z* range. | Structural information (identification) of unknowns via MS^2^ experiments. Background ions are excluded. |

To further validate DART and reagentless DESI experiments, DESI MS was also performed in “reactive” mode [22]. This mode allows for the specific and sensitive detection of artemisinin derivatives. Reactive DESI experiments were performed by adding 100 μM dodecylamine (DDA) (Sigma-Aldrich) to the spray solution, and repeating the procedure described above.
