# Supplementary material for: A Collaborative Epidemiological Investigation into the Criminal Fake Artesunate Trade in South East Asia
Source: PLoS Med. 2008 Feb 12;5(2):e32. doi: 10.1371/journal.pmed.0050032 (PMC2235893; doi:10.1371/journal.pmed.0050032)
Supplement: Supplementary file 4 [file pmed.0050032.sd003.doc]

**Protocol S3. X-ray diffractometry**

X-ray diffractometry (XRD; X'Pert Pro X-ray diffractometer, Philips, Almelo) and isotope ratio analysis were used to determine the mineral composition. Each sample was gently crushed together with a small volume of absolute ethanol, in an agate mortar and pestle and mounted as slurry on a low-background silicon plate and X-rayed as a random mount. When calcite was detected, oxygen and carbon for stable isotope ratio mass spectrometry were extracted [26]. Values, in per mil (‰), are reported in δ^18^O and δ^13^C notation relative to Vienna Standard Mean Ocean Water (VSMOW) and Vienna Peedee Belemnite standard (VPDB), respectively [27]. Data were normalized to the international calcite standard NBS-19. Two analyses of NBS-19 calcite had values that varied by less than 0.1 ‰ for δ^18^O and for δ^13^C. Samples were analysed in duplicate and values averaged.
