# Supplementary material for: A Collaborative Epidemiological Investigation into the Criminal Fake Artesunate Trade in South East Asia
Source: PLoS Med. 2008 Feb 12;5(2):e32. doi: 10.1371/journal.pmed.0050032 (PMC2235893; doi:10.1371/journal.pmed.0050032)
Supplement: Supplementary file 5 [file pmed.0050032.sd004.doc]

**Protocol S4. Botanical analysis**

Most, but not all, spores and pollen are acid-resistant. Tablets containing sucrose were dissolved in hot distilled water, those containing calcite were dissolved in heated 10% hydrochloric acid and those containing talc were mixed in sodium polytungstate (SPT; specific gravity 2.0) to remove the talc as this is resistant to hydrochloric acid. This gave a very good separation with two distinct fractions, both white in colour. The top fraction, where the rare spores and pollen were concentrated, and the tablets composed of starch were then boiled in an acetolysis solution (9 parts acetic anhydride to 1 part sulphuric acid by volume) for several minutes. The genuine starch samples dissolved readily but the counterfeit starch samples were a complex mixture, which did not dissolve easily. They were difficult to process as full elimination of starch would have been detrimental to full spore/pollen recovery. Starch was therefore acetolysed to decrease the size of each granule, with staining of organic material with saffron red so that plant debris could be detected between the starch and remaining talc granules. Any mineral material present was removed by 50% hydrofluoric acid or more effectively by SPT. The abundant fine detritus that remained was removed by filtering through 6 μm mesh. Chemicals were made up to the required strength each time and filtered to eliminate any possible pollen contamination. All residues were mounted in glycerine jelly and placed on glass microscope slides under a long cover slip. The number of slides prepared depended on the amount of residue, there being up to 13 slides per sample. A blank glycerine jelly covered slide exposed to test for possible laboratory contamination during the processing of the samples contained only very rare dust particles, a hair and some fibres off the filter. A New Zealand derived contaminant (*Nothofagus* – southern beech) was found in two samples, but such contaminants are easy to detect as they are more highly stained and contain fully dilated cell contents.
