# Supplementary material for: A Collaborative Epidemiological Investigation into the Criminal Fake Artesunate Trade in South East Asia
Source: PLoS Med. 2008 Feb 12;5(2):e32. doi: 10.1371/journal.pmed.0050032 (PMC2235893; doi:10.1371/journal.pmed.0050032)
Supplement: Supplementary file 6 [file pmed.0050032.st001.doc]

**SUPPLEMENTARY MATERIAL**

**Table S1. Summary of packaging, chemical and botanical analysis of ‘artesunate’ samples. Samples judged to be counterfeit on the basis of packaging characteristics, blinded to the results of chemical analysis, are given in bold.**

| **Samples assessed, with palynology and XRD in the Jupiter investigation 2005-2006** | | | | | | | | | | | |
| --- | --- | --- | --- | --- | --- | --- | --- | --- | --- | --- | --- |
| **Sample** | | **Batch No**  **Mfg Date**  **Exp Date** | | **Hologram**  **and type distribution** | | | | **HPLC mg/tablet** | **Mass spectrometry** | **XRD** | **Botany** |
| Genuine, Guilin Pharmaceutical Co. Ltd. | | 010401  04/01  04/04 | | Genuine | | | | 45 mg artesunate | Artesunate on and in sample | Starch | Cellular plant material, broken tricolporate and Poaceae pollen with rare fungal & bryophyte spores |
| 1/1, Genuine, Guilin Pharmaceutical Co. Ltd. Bought in Ho Chi Minh City, Viet Nam | | 990302  03/99  03/02 | | Genuine | | | | 48 mg artesunate | Artesunate and artemisinin on and in sample | Starch, sucrose | Charcoal, fungal material, very few pollen grains, *Pinus* (50% of pollen), Poaceae and Taxodiaceae pollen |
| S18, Genuine, Guilin Pharmaceutical Co. Ltd. Bought Myanmar/Thai border | | 031102  11/03  11/2007 | | Genuine, with gold foil and ‘Myanmar Reg No’ | | | | 48 mg of artesunate ^1^ | Artesunate on and in sample ^1^ | Starch, sucrose, a little kaolinite | Charcoal, fungal hyphae, very few pollen grains, *Pyrrosia* spores*, Pinus, Ulmus*, Cyperaceae, *Juglans*, and Malvaceae pollen |
| 05/23, Genuine, Mekophar, Hanoi, Viet Nam. Direct from company | | 10105  24/01/05  01/2008 | | No hologram is used with the genuine product | | | | 50 mg artesunate | Artesunate on and in sample | Lactose, starch, magnesium phosphate | Charcoal, black and green synthetic fibres, fungal hyphae and spores, and cellular plant debris some with preserved cell contents. *Pinus, Alnus, Artemisia*, *Betula*, *Corylus*, *Salix*, *Ulmus*, Poaceae, Asteraceae, Chenopodiaceae or Amaranthaceae pollen |
|  |  | Notable for absence of fern spores | | | | | | | | | |
| 05/22, Genuine,  TRAPHACO, Hanoi, Viet Nam, Bought in Laos | | 01 012008  ---  1/1/2008 | | | No hologram is used with the genuine product | | 48 mg artesunate | | Artesunate and traces of artemisinin on and in sample | Lactose, starch | Large charcoal flakes (0.5 mm diameter), blue synthetic fibres, very sparse pollen – *Pinus, Artemisia* and Poaceae pollen |
|  | |  | | |  | |  | |  |  |  |
| **S1**  **Myanmar/**  **Thai border** | | 04010  01/04  01/07 | | | 9 | | 12 mg artesunate/tablet and 4.9% paracetamol^2^ | | Paracetamol ^3^ | Starch, talc, chlorite, quartz | Fungal material, charcoal, hairs, invertebrate remains, very rare pollen and spores. *Abies*, *Pinus*, Cyatheaceae, Myrtaceae, Poaceae pollen and fern spores |
|  |  | Similar flora to 2/15030-34. Batch number on box and blisterpack 040101 and 04010, respectively. Fake insert | | | | | | | | | |
| **S5**  **northern Myanmar** | | 040301  03/04  03/07 | 10 blisterpack in Type 4 packet | | | | <1 mg artesunate/tablet | | Metamizole & paracetamol in sample^3^ | Starch, talc, chlorite, metamizole, decanoic acid^5^ | Charcoal, hairs, invertebrate remains, Very rare pollen and spores. *Alsophila*, Cyatheaceae and fungal spores, *Pinus*, Asteraceae, Chenopodiaceae or Amaranthaceae, Poaceae pollen |
|  |  | Similar flora to S1 and 2/150303-34 | | | | | | | | | |
| **1/2**  **Ho Chi Minh City, Viet Nam** | | 980502  05/98  05/01 | 2 | | | | Artesunate not detected | | No pharmaceuticals identified | Calcite 100%, trace aragonite. δ^13^C -6.79, δ^18^O +13.8 ‰ | Charcoal, cellular plant material, hairs, unidentified invertebrate remains, Polypodiaceae and bryophyte spores, *Pinus*, Curcubitaceae, *Heliotropium*, Myrtaceae, *Plumbago*, Poaceae, *Restio* or *Centrolepis* (rush) pollen |
|  |  | Seasonally dry, irrigated tropical SE Asia, *Pinus* suggests mountains of SE Asia but may drift long distances | | | | | | | | | |
| **1/13**  **northern Myanmar** | | 990308  03/99  03/02 | 1 | | | | Artesunate not detected | | No pharmaceuticals detected^4^ | Starch, talc, sucrose | Charcoal, rare cellular plant material and hairs, *Ulmus* and Poaceae pollen, bryophyte spores, fungal hyphae, tricolpate grains |
|  |  | Starch, talc & sucrose. *Ulmus* suggests mountains of tropical Asia, northern Myanmar-Thailand-Viet Nam and borders with China and northern China | | | | | | | | | |
| **2221**  **southern Laos** | | 010401  04/01  04/04 | 5 | | | | Artesunate not detected | | Paracetamol^3^ | Calcite 20%, starch, sucrose, paracetamol. δ^13^C -9.60,δ^18^O +5.9 ‰ | Charcoal, cellular plant material, no pollen or spores |
|  |  |  | | | | | | | | | |
| **2226**  **southern Laos** | | 010401  04/01  04/04 | 8 | | | | Artesunate not detected | | Paracetamol, dimethylfumarate, metamizole ^3,6^ | Calcite 82%, starch, talc. δ^13^C -9.62, δ^18^O + 7.9 ‰ | Charcoal. Parenchymatous cells similar to 1/2, fungal material and hairs. No pollen or spores |
|  |  | Possibly same source as 1/2. | | | | | | | | | |
| **2242**  **southern Laos** | | 010401  04/01  04/04 | 8 | | | | Artesunate not detected | | Sulphadoxine, chloroquine, chloramphenicol, metamizole | Calcite 80%, starch, talc. δ^13^C -9.57, δ^18^O 8.7 ‰ | Charcoal. Parenchymatous cells similar to 1/2, fungal hyphae, invertebrate remains and hairs. *Alsophila* spores and *Pinus* pollen |
|  |  | Possibly same source as 1/2. | | | | | | | | | |
| **2267**  **southern Laos** | | 000902  09/00  09/03 | 3 | | | | Artesunate not detected | | Dimethylfumarate^3,6^, 2-mercaptobenzothiazole ^3,7^ and erucamide ^3,8^ | Starch, talc | Numerous fine structureless organic particles, fungal hyphae and charcoal. A 5 mm long woody strand. *Artemisia*, *Ulmus* and *Casuarina* or *Carya* pollen. |
|  |  | Seasonally dry SE Asia from northern Thailand to northern Viet Nam. Possibly southern China if *Carya*. | | | | | | | | | |
| **2/12012**  **Vientiane, Laos** | | 010101  01/01  01/04 | 6 | | | | Data not available | | Artemisinin on and in tablet | Acrylamide, starch | Blue synthetic fibres, charcoal, very few pollen grains – *Pinus, Jasminum,* Poaceae, Taxodiaceae pollen |
|  |  |  | | | | | | | | | |
| **2/12051**  **southern Laos** | | 990302  09/00  09/03 | 3 | | | | Artesunate not detected | | 2-mercaptobenzothiazole ^3,7^ | Starch & talc | Charcoal, cellular plant material, *Artemisia*, *Alnus*, *Pinus*, *Salix,* Poaceae and Taxodiaceae (?*Cephalotaxus*) pollen, Dryopteridaceae fern and fungal spores |
|  | | Temperate Asia. *Cephalotaxus* occurs in southern China | | | | | | | | | |
| **2/15030**  **Ho Chi Minh City, Viet Nam** | | 010401  04/01  04/04 | 11 | | | | Artesunate not detected | | Dimethylfumarate^6^ on and in sample | Calcite 81%, aragonite trace, starch. δ^13^C -8.21, δ^18^O +9.9 ‰ | Charcoal, fungal spores & hyphae, leaf cuticles, cellular plant material, almost complete *Dermatophagoides* mite nymph. *Alsophila* tree ferns, Hymenophyllaceae filmy ferns, Polypodiaceae spores & *Abies*, *Taxodium*, *Acer*, *Artemisia*, Asteraceae, Cyperaceae, *Nymphoides*, Poaceae, *Plantago* pollen |
|  |  | Near or downwind from coniferous forest in inland mountainous area of at least moderate rainfall near rice fields | | | | | | | | | |
| **2/15031**  **Ho Chi Minh City, Viet Nam** | | 010401  04/01  04/04 | 11 | | | | Artesunate not detected | | Sulfadoxine, pyrimethamine & dimethylfumarate^6^ on sample. Sulfadoxine & dimethylfumarate in sample | Calcite 81%, aragonite, starch, talc | Parenchymatous cells, *Alsophila, Pinus, Alnus, Pterocarya*, Polypodiaceae, Asteraceae, Cyperaceae, Moraceae, Poaceae, Sapotaceae pollen and fern spores. ~75% of spores and pollen represent grass, pine, ferns and daisies |
|  |  | Same environment as 2/15030. | | | | | | | | | |
| **2/15032**  **Ho Chi Minh City, Viet Nam** | | 010401  04/01  04/04 | 11 | | | | Artesunate not detected | | Dimethylfumarate^6^ and paracetamol on and in sample | Calcite 81%, aragonite, starch, talc | Parenchymatous cells, *Pinus*, *Alsophila, Isoetes, Acacia, Lathyris,* Taxodiaceae, Pteridaceae, Asteraceae, Chenopodiaceae or Amaranthaceae, Labiatae, Myrtaceae, Poaceae pollen and fern spores. ~75% of spores and pollen represent grass, pine, ferns and daisies |
|  |  | Same environment as 2/15030 and 2/15031. Chenopodiaceae common in China and not in SE Asia | | | | | | | | | |
| **2/15033**  **Ho Chi Minh City, Viet Nam** | | 010401  04/01  04/04 | 11 | | | | Artesunate not detected | | Sulphadoxine, pyrimethamine & dimethylfumarate^6^ on and in sample. Traces of erythromycin in sample Trace paracetamol on sample. | Calcite 81%, aragonite, starch, talc | *Pteris cadieri*, Cyatheaceae and Bryophyta spores, *Pinus*, *Alnus*, *Artemisia*, Asteraceae, Chenopodiaceae or Amaranthaceae, Liliaceae, Moraceae, Myrtaceae, Poaceae pollen |
|  |  | Same environment as 2/15030, 2/15031 & 2/15032. Chenopodiaceae common in China and not in SE Asia | | | | | | | | | |
| **2/15034**  **Ho Chi Minh City, Viet Nam** | | 010401  04/01  04/04 | 11 | | | | Artesunate not detected | | Sulphadoxine, pyrimethamine on sample. Sulfadoxine, pyrimethamine, erucamide^8^, erythromycin traces & dimethylfumarate^6^ in sample | Calcite 81%, aragonite, starch, talc | Cyatheaceae spores, *Pinus*, Asteraceae, Chenopodiaceae or Amaranthaceae, Cyperaceae, Myrtaceae, Poaceae pollen |
|  |  | Same environment as 2/15030, 2/15031, 2/1503 & 2/15033. Chenopodiaceae common in China and not in SE Asia | | | | | | | | | |
| **2/12056**  **southern Laos** | | 010901  09/01  09/04 | 4 | | | | Artesunate not detected | | 2-Mercaptobenzothiazole ^3,7^ | Talc, calcite 0.5%. δ^13^C -11.1, δ^18^O, +9.3‰ | Charcoal, very sparse pollen grains – *Pterocarya*, Betulaceae, Rubiaceae pollen and fungal spores |
|  |  | Tablet spelt ‘Tablte’ on blisterpack | | | | | | | | | |
| **05/17**  **southern Laos** | | 040201  02/04  02/07 | 12 | | | | Artesunate not detected | | Dimethylfumarate^6^ on surface | Lactose, starch, calcite 8.5%. δ^13^C 1.9, δ^18^O +24.1 ‰ | Charcoal, clumped thin walled cells with outer “perispore”, possibly of algal origin, very few pollen grains – Betulaceae, Poaceae, Sapindaceae pollen and fungal spores |
|  |  | ‘MFP’ and ‘EXP’ on blisterpack in capitals. XRD suggests marine origin of calcite | | | | | | | | | |
| **1/29**  **Ho Chi Minh City, Viet Nam** | | 980502  05/98  05/01 | 2 | | | | Artesunate not detected | | No pharmaceuticals detected^4^ | Calcite 89.1%. δ^13^C -8.5, δ^18^O +11.2‰ | Charcoal, blue synthetic fibres, fungal hyphae and spores, woody fragments, sparse pollen and spores- *Pinus, Plantago*, *Ulmus*, *Acacia*, Cyatheaceae, Taxodiaceae, Asteraceae, Poaceae |
|  |  | Pollen consistent with northern temperate rather than tropical source. *Acacia* suggests southern China and *Ulmus* the border of China with Myanmar, Thailand and Viet Nam and southern and central temperate China | | | | | | | | | |
| **Samples, seized by the Chinese authorities, assessed with palynology and XRD in 2007** | | | | | | | | | | | |
| **China 07/01** | 021001  10/02  10/05 | | Type 15 | | | 0 artesunate  14.76 artemisinin | | | Artemisinin on and in sample. | talc with minor chlorite | *Alsophila* spores, Asteraceae pollen |
| **China 07/02** | 021001  10/02  10/05 | | Type 15 | | | 0 artesunate  14.50 artemisinin | | | Artemisinin on and in sample. | talc with minor chlorite | *Alsophila* spores, Rubiaceae, Poaceae, Asteraceae pollen and fungal spores |
| **China 07/03** | 021001  10/02  10/05 | | Type 15 | | | 0 artesunate  14.62 artemisinin | | | Artemisinin on and in sample. | talc with minor chlorite | Asteraceae, Lamiaceae pollen |
| China 07/04 | 050609  06/05  06/08 | | Genuine | | | 48.55 artesunate | | | Artesunate on and in sample. | Starch | *Pinus* pollen and fungal spores |
| **China 07/05** | 021001  10/02  10/05 | | Type 15 | | | 0 artesunate  14.07 artemisinin | | | Artemisinin on and in sample. | talc with minor chlorite | *Pinus*, Asteraceae, *Alangium* pollen and monolete spores |
| China 07/06 | 050609  06/05  06/08 | | Genuine | | | 47.96 artesunate | | | Artesunate on and in sample. | Starch | *Carya, Plantago* pollen and fungal hyphae |
| China 07/07 | 050609  06/05  06/08 | | Genuine | | | 48.37 artesunate | | | Artesunate on and in sample. | Starch | Fabaceae, ?*Quercus*, *Carya*, Poaceae pollen and monolete and fungal spores with hyphae |
| China 07/08 | 050609  06/05  06/08 | | Genuine | | | 47.99 artesunate | | | Artesunate on and in sample. | Starch | ?*Tamarindus*, ?Poaceae pollen and monolete spore (?Polypodiaceae), coloured (blue, green and red) and colourless artificial threads |
| China 07/09 | 050609  06/05  06/08 | | Genuine | | | 48.52 artesunate | | | Artesunate on and in sample. | Starch | Labiatae pollen and monolete spores, coloured (blue, brown, green and red) and colourless artificial threads |
| China 07/10 | 050609  06/05  06/08 | | Genuine | | | 46.81 artesunate | | | Artesunate on and in sample. | Starch | *Pinus* pollen and a few, isolated, large, brown fungal spores, coloured (red and blue) and colourless threads |
| **China 07/11** | 021001  10/02  10/05 | | Type 15 | | | 0 artesunate  15.09 artemisinin | | | Artemisinin on and in sample. | talc with minor chlorite | *Alsophila* & monolete spores, *Pinus*, Chenopodiaceae, Asteraceae pollen and short, blue threads |

**Abbreviations:** XRD = X-ray diffractometry.

Charcoal = black chips from vehicle exhausts and fires, Poaceae (grass) pollen are probably rice *Oryza sativa* or sugar cane (*Saccharum*), the hairs appeared to be mammalian hairs.

^1^ All S18 was utilised in botanical investigations. The HPLC, DART and DESI analysis is from a physically identical package bought from the same stock.

^2^ Paracetamol content of a partial tablet was 4.9% by weight.

^3^ These samples had been made into powder – it was therefore not possible to determine whether the active ingredients were present only on the surface of the tablet.

^4^ These two samples were only examined for paracetamol and erythromycin

^5^ Commonly known as capric acid, derived from animal fats and used in manufacture of artificial fruit flavours and perfumes.

^6^ An unknown with elemental formula C_6_H_8_O_4_ was matched to the experimental mass spec data. The identification of dimethylfumarate is

provisional. It is used in the treatment of psoriasis and as an anti-fungal additive to food.

^7^ 2-Mercaptobenzothiazole is a toxic carcinogenic combustible chemical used in pesticides and as a rubber vulcanization agent.

^8^ Erucamide is derived from erucic acid, a monounsaturated omega-9 fatty acid, and used in the manufacture of plastic films in pharmaceutical and food packaging as a slip agent
