# Supplementary material for: A Collaborative Epidemiological Investigation into the Criminal Fake Artesunate Trade in South East Asia
Source: PLoS Med. 2008 Feb 12;5(2):e32. doi: 10.1371/journal.pmed.0050032 (PMC2235893; doi:10.1371/journal.pmed.0050032)
Supplement: Supplementary file 7 [file pmed.0050032.st002.doc]

**Table S2. Description of different types of counterfeit artesunate according to counterfeit hologram type for whole data set.** Stickers – plain text and holograms – bold text. Includes data published in Hall *et al.* (2006), Fernández *et al.* (2006), Nyadong *et al.* (2007) and Newton *et al*. (2006a).

| **Type** | **Packaging** | **Chemistry** | **Botany** | **Where found** |
| --- | --- | --- | --- | --- |
| **1** | No hologram, poor printing of mfg and exp dates | Starch, talc, sucrose  Paracetamol, chloroquine, 2-mercaptobenzothiazole | Charcoal, rare cellular plant material and hairs, *Ulmus* and Poaceae pollen, bryophyte spores, fungal hyphae, tricolpate grains | Viet Nam, Myanmar, Thai/Myanmar border, Northern Laos, Cambodia |
| **2**  970901  980201  980502 | Foil sticker as poor copy of hologram | No artesunate detected  Calcite 100%, trace aragonite. δ^13^C -6.79, δ^18^O +13.8 ‰  Calcite 89.1%. δ^13^C -8.5, δ^18^O +11.2 ‰ | Charcoal, cellular plant material, hairs, an unidentified invertebrate, bryophyte & Polypodiaceae spores, *Pinus*, Curcubitaceae, *Heliotropium*, Myrtaceae, *Plumbago*, Poaceae, *Restio* or *Centrolepis* pollen  Charcoal, blue synthetic fibres, fungal hyphae and spores, woody fragments, sparse pollen & spores - *Pinus, Plantago*, *Ulmus*, *Acacia*, Cyatheaceae, Taxodiaceae, Asteraceae, Poaceae | Viet Nam, Cambodia |
| **3**  **000902** | **Hologram with error in mountain silhouette and pictograms** | **Starch and talc**  **dimethylfumarate,**  **2-mercaptobenzothiazole, erucamide** | **Numerous fine structureless organic particles, fungal hyphae and charcoal. A 5 mm long woody strand. *Artemisia*, *Ulmus* and *Casuarina* or *Carya* pollen.**  **Charcoal, cellular plant material, fungal spores. *Artemisia*, *Alnus*, *Pinus*, *Salix,* Poaceae and Taxodiaceae (?*Cephalotaxus*) pollen, Dryopteridaceae fern spores** | **Southern Laos, Cambodia** |
| **4**  **010901**  **021001**  **020305** | **Good hologram copy but lacks microscopic ‘Guilin Pharma’ legend and has ‘Tablte’ printed on blisterpack** | **Talc and calcite 0.5%. δ^13^C -11.1, δ^18^O, +9.3 ‰**  **2-mercaptobenzothiazole, safrole, paracetamol, metamizole, artemisinin, artesunate, dimethylfumarate** | **Charcoal, very sparse pollen grains – *Pterocarya*, Betulaceae, Rubiaceae & fungal spores** | **Myanmar, southern Laos** |
| **5**  010401 | Foil sticker made of thousands of dots. | Calcite 20%, starch, sucrose, δ^13^C -9.60,δ^18^O +5.9 ‰  Sulfadoxine, pyrimethamine, dimethylfumarate, erythromycin, paracetamol, metamizole, artemisinin | Charcoal, cellular plant material, no pollen or spores | Southern Laos |
| **6**  **030101**  **010101** | **Hologram, mountains/waves junction differs, an indistinct legend to the right of centre,**  **beneath the waves** | **Acrylamide, starch**  **Chloramenphicol, metronidazole, artemisinin.** | **Blue synthetic fibres, charcoal, very few pollen grains – *Pinus, Jasminum,* Poaceae, Taxodiaceae** | **Northern Laos** |
| **7**  **000801** | **Hologram. ‘Guilin Pharma’ legend present but in larger font and can be read with the naked eye. Date of expiry 9 years after manufacture** | **artemisinin** | **----------** | **Southern Laos** |
| **8**  980201  010401  040201 | Poor sticker with shoulder plateau on mountain. Abbreviations in capital letters ‘MFG’, ‘EXP’ | Artesunate not detected  Calcite 90-82%, starch, talc. δ^13^C -9.62, δ^18^O + 7.9 ‰ and δ^13^C -9.57, δ^18^O 8.7 ‰  Sulfadoxine, pyrimethamine, erucamide, dimethylfumarate, erythromycin, safrole,  paracetamol, metamizole, artemisinin, chloroquine, chloramphenicol | Charcoal. Parenchymatous cells similar to Type 2, fungal material and hairs. No pollen or spores  Charcoal. Parenchymatous cells similar to Type 2, fungal hyphae, invertebrate remains and hairs. *Alsophila* spores and *Pinus* pollen | Southern and northern Laos, Viet Nam |
| **9**  **040101**  **4010**  **040502** | **Good hologram with ‘Guilin Pharma’ legend in correct font** | **Starch, talc, chlorite and quartz**  **Paracetamol, safrole, traces of artesunate (~10mg/tablet)** | **Fungal material, charcoal, hairs, invertebrate remains, very rare pollen and spores-*Abies*, *Pinus*, Cyatheaceae, Myrtaceae, Poaceae** | **Myanmar, Thai/Myanmar border** |
| **10**  **040301** | **Hologram, similar to ‘Type 4’, but has ‘X-52’ visible under**  **UV light on hologram** | **Starch, talc, chlorite,**  **metamizole, decacoic acid, artesunate, paracetamol, safrole, dimethylfumarate** | **Charcoal, hairs, invertebrate remains, fungal spores. Very rare pollen and fern spores-*Alsophila*, *Pinus*, Cyatheaceae, Asteraceae, Chenopodiaceae or Amaranthaceae, Poaceae** | **Myanmar, northern Laos** |
| **11**  010401  020402  030301 | Sticker, similar to Types 2 &  8 but mountain outline differs. Abbreviations in capital letters ‘MFG’, ‘EXP’ | Calcite 81%, aragonite trace, starch. δ^13^C -8.21, δ^18^O +9.9 ‰ and δ^13^C -8.21, δ^18^O +9.9 ‰  Sulfadoxine, pyrimethamine, erucamide, dimethylfumarate,  erythromycin, safrole, paracetamol, artemisinin | Charcoal, fungal spores & hyphae, leaf cuticles, cellular plant material, *Dermatophagoides* mite nymph. *Alsophila* ferns, Hymenophyllaceae filmy ferns, Polypodiaceae, *Abies*, *Taxodium*, *Acer*, *Artemisia*, Asteraceae, Cyperaceae, *Nymphoides*, Poaceae, *Plantago*  Parenchymatous cells, *Alsophila, Pinus, Alnus, Pterocarya*, Polypodiaceae, Asteraceae, Cyperaceae, Moraceae, Poaceae, Sapotaceae pollen and fern spores. ~75% of spores and pollen represent grass, pine, ferns and daisies  Parenchymatous cells, *Pinus*, *Alsophila, Isoetes, Acacia, Lathyris,* Taxodiaceae, Pteridaceae, Asteraceae, Chenopodiaceae or Amaranthaceae, Labiatae, Myrtaceae, Poaceae pollen and fern spores. ~75% of spores and pollen represent grass, pine, ferns and daisies  *Pinus*, *Alnus*, *Artemisia*, Cyatheaceae & *Pteris cadieri* spores, Asteraceae, Chenopodiaceae or Amaranthaceae, Liliaceae, Moraceae, Myrtaceae, Poaceae pollen and Bryophyta spores  Cyatheaceae spores*, Pinus*, Asteraceae, Chenopodiaceae or Amaranthaceae, Cyperaceae, Myrtaceae, Poaceae pollen | Southern Laos, Viet Nam |
| **12**  040201 | Sticker, with similar mountain outline to Types 8 & 11 but with a ‘cloth’ like appearance. Abbreviations in capital letters ‘MFG’, ‘EXP’ | Lactose, starch, calcite 8.5%. δ^13^C 1.9, δ^18^O +24.1 ‰  Erucamide, dimethylfumarate, safrole | Charcoal, clumped thin walled cells with outer “perispore”, possibly of algal origin, very few pollen grains – Betulaceae, Poaceae, Sapindaceae, fungal spores | Southern Laos |
| **13**  **04010** | **Hologram, with similar mountain outline to Types 4 & 9 but with different pictograms and outer circle. No ‘Gulin Pharma’ legend** | **----------------** | **-------------------** | **Myanmar** |
| **14**  **050701** | **Hologram with inner and outer circles misaligned and has ‘Myanmar Reg No’ on blisterpack** | **Traces of artesunate on tablet surface** | **-------------------** | **Myanmar** |
| **15**  **021001** | **Hologram similar to Type 10 with same misaligned blocks. No ‘X-52’ visible under UV light** | **Talc with minor chlorite Artemisinin** | ***Pinus*, *Alangium*, Rubiaceae, Poaceae, Asteraceae pollen & fungal *Alsophila*, and monolete spores** | **China** |

See Figures 2 & S1 for illustrations and descriptions of counterfeit holograms and stickers.

All counterfeit holograms have mis-registered outer rings of curved blocks.
